# Strategies for care quality improvement in Cystic Fibrosis

**DOI:** 10.1186/s13023-017-0743-9

**Published:** 2018-02-08

**Authors:** Gilles Rault, Pierre Lombrail

**Affiliations:** 1 CF Center, Fondation ildys, Roscoff, France; 2 LEPS EA3412, Sorbonne Paris Cite University, Bobigny, France

Cystic fibrosis (CF) is a “model” of international collaboration for therapeutic research, social science research, development of international guidelines and care management all at once, because of its characteristics: a genetic disease which is progressive, chronic and multisystemic, with a prevailing impairment of the respiratory function, and also a “rare disease”, albeit the most common of “rare diseases” in Caucasian populations.

Globally, the 1980s were marked by the first successful pulmonary transplant on cystic fibrosis patients and the discovery of the *CFTR* gene. “Resignation” gave way to hope, based on the acceleration of research efforts shown by the simultaneous increase of articles on this disease.

In France, a greater interest for this disease from medical teams, a better care management by multidisciplinary teams in specialized health centres and the creation of the National Cystic Fibrosis Observatory (1992) marked this turning point. In the early 2000s, the national application of systematic neonatal CF screening led to a structuring characterised by the recognition by the health authorities of Cystic Fibrosis Centres (CFCs) (2002) meeting the criteria of CF care specifications. In the frame of the National Plan for Rare Diseases, 2 expertise centres for CF (CF-CERD) were certified in 2006 and the CFTR care sector was identified (2014).

The implementation of the PHARE-M care quality improvement program (QIP) (*‘A hospital-based program for improvement of results and expertise in cystic fibrosis care’)* is the logical, yet pioneer, extension of the care sector structuring for this rare disease. The PHARE-M puts forward a major development to bring interdisciplinarity at the center of the teams’ practice and to strengthen the partnership with patients and parents to improve patient care at their CFC.

Indeed, this quality approach targets the clinical microsystem, which includes the CFC professional team, patients and their relatives, and professionals in the city involved in care, because the health results and the patient’s quality of life depend on the functioning of the overall system [1]:

- in a systemic vision of the care production process (the care manufacture): “*a system is perfectly designed to produce the results it produces*”
- and the assertion of the interdependency of the various links: “*no one is solely responsible for the results, whatever they are*”

This cultural evolution is supported by a collaborative dynamic and requires an ethics of cooperation that enables exchanges between CFCs on their results and on the “potential best practices” identified through benchmarking. It is perpetuated through the implementation of measuring tools that allow to follow the results of the actions undertaken and the facilitation of a community that exchanges on continuous quality improvement. It is the subject of research on prevention and healthcare services, a token of continuous improvement of care quality founded on “evidence-based” data.

## What was the genesis of the PHARE-M QIP in cystic fibrosis in France?

The PHARE-M program relies on the success of the American experience hailed by an article in the Thorax journal in August 2011 [2]. The triggering event that occurred ten years earlier was the publication by the US Institute of Medicine of the article entitled “Crossing the quality chasm: a new health system for the 21st century” [3].

Immediately following this publication, the American Cystic Fibrosis Foundation (US CFF) called upon the services of experts from the Institute for Healthcare Improvement (IHI, Harvard) and The Dartmouth Institute Microsystem Academy (TDIMA). It then observed a great disparity of survival results from one center to the next, based on the indicators found in the US Cystic Fibrosis Registry; it organised a benchmarking visit of the 10 “best” centers to identify the key success factors; it decided to release with full transparency the results indicators for the various centers; and it made the decision to establish a Care Quality Improvement Program in the United States.

From 2002 to 2013, the CFF organized, with experts from the TDIMA, annual collaborative sessions under the program and gradually tailored this latter to the specificities of cystic fibrosis care management in the USA [4]. The special May 2014 issue of the BMJ Quality & Safety journal entitled “Ten years of improvement: innovation in cystic fibrosis care” [5] recounts in detail that experience and the results achieved.

From 2008 onwards, close ties developed between the Nantes-Roscoff CF-CERD, the ‘Vaincre la Mucoviscidose’ association and the US CFF [6]. In September 2011, the CF-CERD launched the PHARE-M program with a pilot phase, involving 7 CFCs representing about 1000 patients out of nearly 6000 patients present in the French Cystic Fibrosis Registry in 2011 [7].

## What can be found in this supplement?

Beyond the origins of the PHARE-M program, the purpose of this special issue is to report on the quality approach implemented since 2012 at the CFCs involved under the PHARE-M program, its standardization in the landscape of continuing hospital training and the results observed in 2015 after three years of ongoing work. These articles therefore contribute to introducing this intervention in various clinical microsystems and concern different sectors of cystic fibrosis care, nutritional care in pediatrics [8], psychosocial care for teenagers [9], as well as the preparation for pulmonary transplant in adults [10].

In December 2012, the ministry selected and funded the PHARE-M Performance research program, which seeks to assess the impact of the PHARE-M on the evolution of patient health indicators and includes a realistic analysis “to understand what works, for whom and under which circumstances” [11]. The description of the research program protocol [12] and the results of the quality controls of data transferred to the Registry conducted for that purpose [13] enable to understand the assessment methods of the PHARE-M quality program performance and identify their limitations. The conclusion seeks to emphasize the contributions of patients and parents to this collaborative program for the improvement of care quality side by side with the teams at their CFC [14].

Despite the difficulties related to the transposition and adoption of such an approach in different cultural and healthcare systems, we can state that this strategy has had a profound impact on the network of CFCs trained in France, with a great satisfaction within the healthcare teams, an improvement of their interdisciplinary practice, the development of patient therapeutic education, and a strengthened collaboration between patients, parents and healthcare staff in improving care, all of the above supported by a constant research endeavour.
